# Monocyte distribution width as a novel sepsis indicator in COVID-19 patients

**DOI:** 10.1186/s12879-021-07016-4

**Published:** 2022-01-04

**Authors:** Laila Alsuwaidi, Saba Al Heialy, Nahid Shaikh, Firas Al Najjar, Rania Seliem, Aaron Han, Mahmood Hachim

**Affiliations:** 1 College of Medicine, Mohammed Bin Rashid University of Medicine and Health Sciences, P.O. Box: 505055, Dubai, UAE; 2 Rashid Hospital, Dubai Health Authority, Dubai, UAE; 3 Meakins-Christie Laboratories, Research Institute of the McGill University Health Center, Montreal, QC Canada; 4 Kings College Hospital London Dubai, Dubai, UAE

**Keywords:** COVID-19, SARS-CoV-2, MDW, Monocyte, Sepsis

## Abstract

**Background:**

The severe acute respiratory syndrome coronavirus (SARS-CoV-2) is a highly transmittable virus which causes the novel coronavirus disease (COVID-19). Monocyte distribution width (MDW) is an in-vitro hematological parameter which describes the changes in monocyte size distribution and can indicate progression from localized infection to systemic infection. In this study we evaluated the correlation between the laboratory parameters and available clinical data in different quartiles of MDW to predict the progression and severity of COVID-19 infection.

**Methods:**

A retrospective analysis of clinical data collected in the Emergency Department of Rashid Hospital Trauma Center-DHA from adult individuals tested for SARS-CoV-2 between January and June 2020. The patients (n = 2454) were assigned into quartiles based on their MDW value on admission. The four groups were analyzed to determine if MDW was an indicator to identify patients who are at increased risk for progression to sepsis.

**Results:**

Our data showed a significant positive correlation between MDW and various laboratory parameters associated with SARS-CoV-2 infection. The study also revealed that MDW ≥ 24.685 has a strong correlation with poor prognosis of COVID-19.

**Conclusions:**

Monitoring of monocytes provides a window into the systemic inflammation caused by infection and can aid in evaluating the progression and severity of COVID-19 infection.

**Supplementary Information:**

The online version contains supplementary material available at 10.1186/s12879-021-07016-4.

## Introduction

The severe acute respiratory syndrome coronavirus (SARS-CoV-2) is a highly transmittable virus which causes the novel coronavirus disease (COVID-19) that has affected over 131 million people worldwide and has caused 2.85 million deaths globally as of April 5th, 2021. The most common clinical presentation of this disease includes fever, dry cough and fatigue. However, in a subset of COVID-19 patients, severe outcomes such as viral sepsis are seen. Sepsis is a life-threatening systemic illness which can result in dysregulated immune responses leading to organ dysfunction and a leading cause of mortality [1].

To date, several biomarkers have been identified as early markers to evaluate inflammation and disease outcomes such as C-reactive protein, creatinine and D-dimer [2]. In response to infection, the first immune cells to be recruited are neutrophils and monocytes. In fact, monocyte distribution width (MDW) is used as a biomarker for sepsis where levels > 20 are indicative of sepsis [3]. MDW is an in-vitro hematological parameter which describes the changes in monocyte size distribution and can indicate progression from localized infection to systemic infection [4]. This parameter can be performed along with other routine parameters on several Beckman Coulter DxH analyzers. MDW alone or in combination with white blood count (WBC) can be used to detect early sepsis in the emergency department [5]. A recent study showed that combining MDW ≥ 20 and Neutrophil-to-lymphocyte ratio (NLR) < 3.2 is more efficient in identifying COVID-19 and can be actually used to distinguish SARS-CoV-2 infection from influenza infection and other upper respiratory tract infections [6]. Monitoring of monocytes provides a window into the systemic inflammation caused by infection and can aid in evaluating the progression and severity of the infection.

In this study, we retrospectively analyzed the clinical and biological characteristics of the COVID-19 infected patients and investigated the ability of MDW to predict at an earlier time the disease severity, in comparison with other biomarkers. We also investigated the correlation between routine laboratory parameters in different quartiles of MDW values to evaluate the usefulness of this value in predicting disease outcomes.

## Materials and methods

### Study population and design

This is a retrospective cohort study, which includes all adult individuals (≥ 18 years old) tested for SARS-CoV-2 in the Emergency Department—Rashid Hospital Trauma Center of DHA between January and June 2020. We included only the laboratory-confirmed cases, as the diagnosis was performed by real-time reverse transcriptase-polymerase chain reaction (RT-PCR) conducted on a nasopharyngeal swab of the patient according to the World Health Organization (WHO) guidance.

Epidemiological characteristics including demographics, recent exposure history, clinical symptoms and signs, and laboratory findings, were obtained from the patients’ electronic medical records in DHA unified electronic system *Salama* using a standardized data collection form, which is a modified version of the WHO/International Severe Acute Respiratory and Emerging Infection Consortium case record form for severe acute respiratory infections (Additional file 1: Appendix 1).

### Clinical and laboratory data

In terms of epidemiological information, we considered patient demographic characteristics including age and gender; clinical symptoms including fever, cough, respiratory symptom, ear, nose and throat symptom; comorbidities including hypertension, diabetes, cardiovascular disease, respiratory disease, and other disease.

Venous blood samples and nasal-pharyngeal swabs were collected and examined by the Emergency Department Laboratory of Rashid Hospital Trauma Center of DHA. Initial investigations included hematological analysis (complete blood count and coagulation profile), serum biochemical test (renal and liver function, creatine kinase, lactate dehydrogenase, electrolytes, and serum ferritin) in addition to some inflammatory markers (procalcitonin and C-Reactive Protein). Frequency of examinations was determined according to the disease progress. For hospitalized patients, nasopharyngeal swab specimens were obtained for SARS-CoV-2 RT-PCR re-examination every other day after clinical remission of symptoms, including fever, cough, and dyspnea. Repeat RT-PCR tests were performed for SARS-CoV-2 done in patients confirmed to have COVID-19 infection to show viral clearance before hospital discharge or discontinuation of isolation as per national guidelines at the time of this study.

The MDW, which was measured in this study using Beckman Coulter DxH 900 analyzer, is an additional parameter that was recorded in the data collection form. MDW values were compared among the studied groups to determine its usefulness as an indicator to identify patients who are at increased risk for progression to sepsis.

### Statistical analysis

Data were presented as mean and standard deviation for continuous variables and frequency (number and percentage; %) for categorical variables. For all statistical analyses and tests, SPSS was used (Released 2019. IBM SPSS Statistics for Windows, Version 26.0. Armonk, NY: IBM Corp). The normality test for all groups was done by Shapiro–Wilk tests using SPSS, and sig. of all independent variables > 0.05 means that all groups were normally distributed. To assess the differences between COVID-19 patients different groups based on MDW, ANOVA: analysis of variance used to identify and compare variances among groups for the continuous variables and Chi-square test was used for the categorical variables. P value < 0.05 had been considered significant.

## Results

**Fig. 1:**
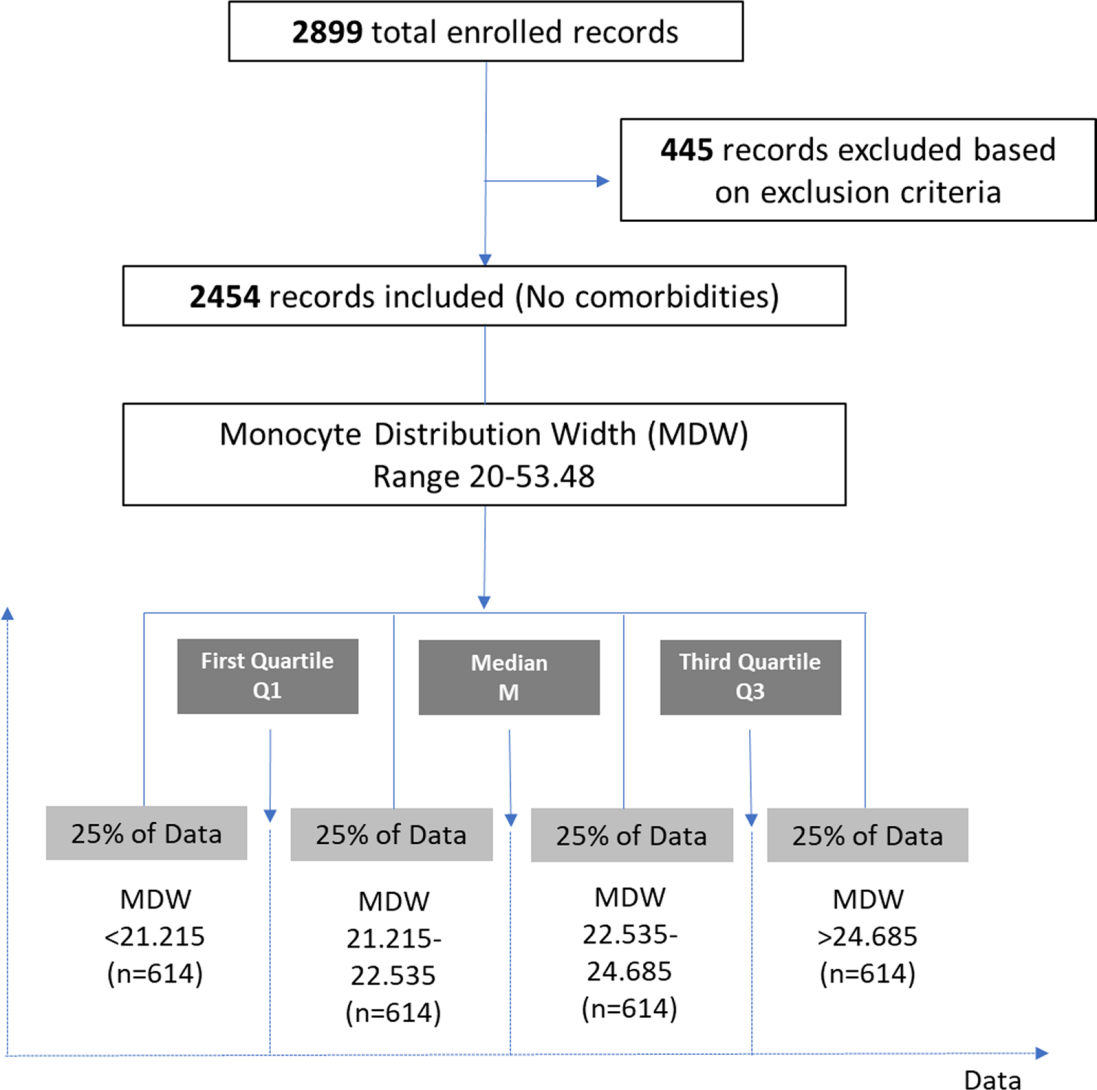
Study design and subject flowchart

From January to June 2020, 2899 patients were tested positive for SARS-CoV-2 in the Emergency Department of Rashid Hospital Trauma Center of DHA. Only positive COVID-19 patients who had no comorbidities were selected for further analysis (n = 2454) as demonstrated in Fig. 1. The age range was 72 (18–90) years, and 78.7% were men. Further characteristics of the studied population are summarized in Table 1.

**Table 1:** Characteristics of the study population

	No	Mean	Std. error of mean	Std. deviation	Skewness	Std. error of skewness	Range	Minimum	Maximum
*Demographics*									
Age (years)	2454	41.54	0.282	13.994	0.777	0.049	72	18	90
*Hematology markers*									
White blood cell (× 10^3^ per μL)	2454	8.082	0.0836	4.1409	2.186	0.049	37.9	1.2	39.1
Platelet (× 10^3^ per μL)	2454	227.71	1.863	92.27	2.095	0.049	1008	7	1015
Neutrophil %	2454	70.059	0.2629	13.0235	− 0.722	0.049	85.7	10.6	96.3
Lymphocyte %	2454	18.919	0.2144	10.6225	1.125	0.049	86	1.1	87.1
Monocyte %	2454	9.764	0.0872	4.3198	0.958	0.049	42.5	1.4	43.9
Neutrophil absolute (× 103 per μL)	2454	5.911	0.0765	3.7892	2.251	0.049	35.2	0.5	35.7
Lymphocyte absolute (× 103 per μL)	2454	1.345	0.0204	1.01	13.219	0.049	31.9	0.1	32
Monocyte absolute (× 103 per μL)	2454	0.728	0.0079	0.3923	2.01	0.049	4.7	0	4.7
Monocyte distribution Width (U)	2454	23.5053	0.07008	3.47177	2.594	0.049	33.48	20	53.48
Coagulation markers									
Prothrombin time (s)	1518	14.31	0.05	1.931	6.718	0.063	37	11	48
APTT (s)	1499	38.97	0.161	6.252	3.633	0.063	99	13	112
D-dimer (μg/mL)	729	1.24	0.069	1.857	5.102	0.091	18	0	18
Fibrinogen (mg/dL)	16	559.88	32.539	130.158	− 0.158	0.564	433	357	790
Troponin (pg/mL)	447	79.77	37.2	786.492	19.126	0.115	16,048	3	16,051
COVID-19 inflammation markers									
C-reactive protein (mg/L)	2276	69.1298	1.74835	83.40931	1.999	0.051	569.1	0.4	569.5
LDH (U/L)	1287	303.47	4.427	158.832	3.68	0.068	2492	6	2498
Ferritin (ng/mL)	1047	849.17	30.249	978.782	4.326	0.076	13,951	9	13,960
Procalcitonin (ng/mL)	1887	1.91744	0.512958	22.28269	29.416	0.056	831.38	0.02	831.4
Liver enzymes									
Albumin (g/dL)	1851	3.8925	0.01282	0.55173	− 0.945	0.057	4.8	0.6	5.4
ALT (U/L)	1855	44.178	1.8189	78.3383	22.22	0.057	2662.8	3.2	2666
AST (U/L)	350	76.12	18.165	339.827	14.614	0.13	5808	0	5808
Bilirubin, total (mg/dL)	1856	0.67	0.022	0.963	18.824	0.057	31	0	31
Creatinine (mg/dL)	2276	1.079	0.0795	3.7931	26.99	0.051	125.8	0.1	125.9
Death	43	43,954.11	3.031601	19.87954	0.379	0.361	68.2875	43,924.94	43,993.23

*APTT* activated partial thromboplastin time, *LDH* lactate dehydrogenase, *ALT* alanine aminotransferase, *AST* aspartate aminotransferase

As presented in Table 2, the correlation between MDW and major hematology laboratory markers used routinely in assessing cases of COVID-19 in an emergency department setting. Pearson Correlation between MDW and all blood results for all patients included in the study (n = 2454) showed that MDW was positively correlated with WBC (r = 0.101, p < 0.001), neutrophils percentage (NE%) (r = 0.250, p < 0.001), neutrophils count (NE#) (r = 0.162, p < 0.001). Nevertheless, significant negative correlation was observed between MDW and total platelet (PLT) (r = − 0.140, p < 0.001), lymphocytes percentage (LY%) (r = − 0.168, p < 0.001), and monocytes percentage (MO%) (r = − 0.262, p < 0.001).

**Table 2:** Correlation between MDW and major laboratory markers used routinely in assessing cases of COVID-19 in an emergency department setting

Correlations		MDW
Age (years)	Pearson correlation	0.065
	Sig. (2-tailed)	0.001
	N	2454
White blood cell (× 10^3^ per μL)	Pearson correlation	0.101
	Sig. (2-tailed)	< 0.001
	N	2454
Neutrophil %	Pearson correlation	0.250
	Sig. (2-tailed)	< 0.001
	N	2454
Lymphocyte %	Pearson correlation	− .168
	Sig. (2-tailed)	< 0.001
	N	2454
Monocyte %	Pearson correlation	− .262
	Sig. (2-tailed)	< 0.001
	N	2454
Neutrophil absolute (× 10^3^ per μL)	Pearson correlation	0.162
	Sig. (2-tailed)	< 0.001
	N	2454
Lymphocyte absolute (× 10^3^ per μL)	Pearson correlation	− .104
	Sig. (2-tailed)	< 0.001
	N	2454
Monocyte absolute (× 10^3^ per μL)	Pearson correlation	− .175
	Sig. (2-tailed)	< 0.001
	N	2454
Platelet (× 10^3^ per μL)	Pearson correlation	− 0.140
	Sig. (2-tailed)	< 0.001
	N	2454

MDW was positively correlated with total WBC and neutrophils and negatively correlated with total platelet, lymphocytes, monocytes

The results of the current study indicated significant positive correlation between MDW and COVID inflammation markers including C-reactive protein (CRP) (r = 0.401, p < 0.001), lactate dehydrogenase (LDH) (r = 0.381, p < 0.001), Ferritin (r = 0.305, p < 0.001), and Procalcitonin (r = 0.133, p < 0.001) as shown in Table 3. Interestingly, MDW was significantly correlated with the prothrombin time (PT) (r = 0.174, p < 0.001), activated partial thromboplastin time (APTT) (r = 0.204, p < 0.001), and D-Dimer (r = − 0.218, p < 0.001) but there was no correlation between MDW and fibrinogen level and Troponin (Table 4). Additionally, MDW was positively correlated with liver enzymes, alanine aminotransferase (ALT) (r = 0.091, p < 0.001), aspartate aminotransferase (AST) (r = 0.115, p < 0.001), and Total Bilirubin (r = 0. 109, p < 0.001). The only negative correlation was between MDW and Serum albumin r = − 0. 322, p < 0.001) (Table 5).

**Table 3:** Correlation between MDW and COVID-19 inflammation markers

Correlations		MDW
C-reactive protein (mg/L)	Pearson correlation	0.401
	Sig. (2-tailed)	< 0.001
	N	2276
LDH (U/L)	Pearson correlation	0.381
	Sig. (2-tailed)	< 0.001
	N	1287
Ferritin (ng/mL)	Pearson correlation	0.305
	Sig. (2-tailed)	< 0.001
	N	1047
Procalcitonin (ng/mL)	Pearson correlation	0.133
	Sig. (2-tailed)	< 0.001
	N	1887

*LDH* lactate dehydrogenase

**Table 4:** Correlation between MDW and coagulation markers

Correlations		MDW
Prothrombin time (s)	Pearson correlation	**0.174**
	Sig. (2-tailed)	< 0.001
	N	1518
APTT (s)	Pearson correlation	0.204
	Sig. (2-tailed)	< 0.001
	N	1499
D-dimer (μg/mL)	Pearson correlation	0.218
	Sig. (2-tailed)	< 0.001
	N	729
Fibrinogen (mg/dL)	Pearson correlation	0.237
	Sig. (2-tailed)	0.377
	N	16
Troponin (pg/mL)	Pearson correlation	− 0.016
	Sig. (2-tailed)	0.732
	N	447

*PT* prothrombin time; *APTT* activated partial thromboplastin time

**Table 5:** Correlation between MDW and liver enzymes

Correlations		MDW
Albumin (g/dL)	Pearson correlation	− 0.322
	Sig. (2-tailed)	< 0.001
	N	1851
ALT (U/L)	Pearson correlation	0.091
	Sig. (2-tailed)	< 0.001
	N	1855
AST (U/L)	Pearson correlation	0.115
	Sig. (2-tailed)	0.031
	N	350
Bilirubin, total (mg/dL)	Pearson correlation	0.109
	Sig. (2-tailed)	< 0.001
	N	1856
Creatinine (mg/dL)	Pearson correlation	0.023
	Sig. (2-tailed)	0.273
	N	2276

*ALB* albumin; *ALT* alanine aminotransferase, *AST* aspartate aminotransferase

Based on the MDW value, the patients were divided into quartiles with approximately equal numbers of patients assigned to each of the four groups as follows: Q1 (MDW < 21.215, n = 614), Q2 (MDW = 21.215–22.535, n = 614), Q3 (MDW = 22.535–24.685, n = 614) and Q4(MDW ≥ 24.685, n = 614) (Fig. 1). Comparing the different blood biomarkers in each MDW quartile showed that patients with MDW ≥ 24.685 (Q4) demonstrated a strong correlation with poor prognosis COVID-19 related biomarkers. Such patients showed significantly lower platelet counts (Q1 = 240.65 ± 101.408, Q2 = 236.4 ± 96.429, Q3 = 223.53 ± 82.662 and Q4 = 210.24 ± 84.356, p < 0.001) and higher neutrophils percentage (Q1 = 66.449 ± 12.8279, Q2 = 67.864 ± 12.6981, Q3 = 70.98 ± 11.8736 and Q4 = 74.946 ± 13.0348, p < 0.001). Likewise, Q4 patients showed lower lymphocytes percentage (Q1 = 21.301 ± 10.9329, Q2 = 19.717 ± 10.5829, Q3 = 18.373 ± 10.0544 and Q4 = 16.284 ± 10.2825, p < 0.001) and monocytes percentage (Q1 = 10.489 ± 4.0981, Q2 = 10.815 ± 4.2217, Q3 = 9.732 ± 4.1094 and Q4 = 8.019 ± 4.307, p < 0.001). Apparently, the results revealed that all inflammatory markers and risk to develop coagulations markers were significantly higher in Q4 patients compared to the rest of patients in different quartiles (Table 6).

**Table 6:** Comparing the different blood biomarkers of COVID-19 patients in each MDW quartile

Parameter	Quartile	N	Mean	Std. deviation	Std. error	Minimum	Maximum	ANOVA
*Hematology markers*								
White blood cell (× 10^3^ per μL)	1	613	8.056	3.999	0.1615	2.2	33.5	0.403
	2	614	7.845	3.6683	0.148	2.1	27.2	
	3	614	7.883	3.7089	0.1497	2.2	36.8	
	4	613	8.544	5.0169	0.2026	1.2	39.1	
	Total	2454	8.082	4.1409	0.0836	1.2	39.1	
Platelet (× 10^3^ per μL)	1	613	240.65	101.408	4.096	77	1015	< 0.001
	2	614	236.4	96.429	3.892	34	980	
	3	614	223.53	82.662	3.336	10	650	
	4	613	210.24	84.356	3.407	7	638	
	Total	2454	227.71	92.27	1.863	7	1015	
Neutrophil %	1	613	66.449	12.8279	0.5181	22.5	96	< 0.001
	2	614	67.864	12.6981	0.5125	19.1	94.8	
	3	614	70.98	11.8736	0.4792	10.6	94	
	4	613	74.946	13.0348	0.5265	18.4	96.3	
	Total	2454	70.059	13.0235	0.2629	10.6	96.3	
Lymphocyte %	1	613	21.301	10.9329	0.4416	2	57.8	< 0.001
	2	614	19.717	10.5829	0.4271	1.6	62.9	
	3	614	18.373	10.0544	0.4058	2	87.1	
	4	613	16.284	10.2825	0.4153	1.1	65.7	
	Total	2454	18.919	10.6225	0.2144	1.1	87.1	
Monocyte %	1	613	10.489	4.0981	0.1655	1.6	26.7	< 0.001
	2	614	10.815	4.2217	0.1704	2.1	43.9	
	3	614	9.732	4.1094	0.1658	1.6	40.5	
	4	613	8.019	4.307	0.174	1.4	32.1	
	Total	2454	9.764	4.3198	0.0872	1.4	43.9	
Neutrophil absolute (× 10^3^ per μL)	1	613	5.628	3.6845	0.1488	0.7	31.4	< 0.001
	2	614	5.569	3.3693	0.136	0.6	25.8	
	3	614	5.751	3.1023	0.1252	0.6	24.3	
	4	613	6.697	4.7034	0.19	0.5	35.7	
	Total	2454	5.911	3.7892	0.0765	0.5	35.7	
Lymphocyte absolute (× 10^3^ per μL)	1	613	1.517	0.8252	0.0333	0.2	6.8	< 0.001
	2	614	1.362	0.6802	0.0275	0.2	4.3	
	3	614	1.336	1.4619	0.059	0.2	32	
	4	613	1.164	0.8607	0.0348	0.1	11.7	
	Total	2454	1.345	1.01	0.0204	0.1	32	
Monocyte absolute (× 10^3^ per μL)	1	613	0.779	0.3538	0.0143	0.2	2.4	< 0.001
	2	614	0.789	0.3908	0.0158	0.2	4.7	
	3	614	0.723	0.3839	0.0155	0.1	3.7	
	4	613	0.621	0.4162	0.0168	0	4.4	
	Total	2454	0.728	0.3923	0.0079	0	4.7	
*Coagulation markers*								
Prothrombin time (s)	1	331	14.13	1.456	0.08	11	27	0.403
	2	356	14.22	1.461	0.077	12	28	
	3	397	14.16	2.153	0.108	12	48	
	4	434	14.64	2.3	0.11	12	32	
	Total	1518	14.31	1.931	0.05	11	48	
APTT (s)	1	327	37.68	4.29	0.237	27	54	< 0.001
	2	348	38.8	6.171	0.331	26	81	
	3	394	38.6	5.844	0.294	13	107	
	4	430	40.42	7.542	0.364	27	112	
	Total	1499	38.97	6.252	0.161	13	112	
D-dimer (μg/mL)	1	146	1.1	1.863	0.154	0	14	< 0.001
	2	148	1.14	2.148	0.177	0	18	
	3	197	0.99	1.146	0.082	0	11	
	4	238	1.59	2.081	0.135	0	18	
	Total	729	1.24	1.857	0.069	0	18	
Troponin (pg/mL)	1	112	176.49	1515.662	143.217	3	16,051	0.403
	2	84	66.21	420.444	45.874	3	3843	
	3	114	25.1	89.552	8.387	3	928	
	4	137	54.5	167.589	14.318	3	1408	
	Total	447	79.77	786.492	37.2	3	16,051	
*COVID-19 inflammation markers*								
Ferritin (ng/mL)	1	215	466.13	477.287	32.551	9	2835	< 0.001
	2	234	616.69	773.147	50.542	9	8018	
	3	280	865.08	793.872	47.443	9	5222	
	4	318	1265.2	1303.864	73.117	41	13,960	
	Total	1047	849.17	978.782	30.249	9	13,960	
LDH (U/L)	1	267	232.35	88.064	5.389	6	748	< 0.001
	2	300	250.94	100.639	5.81	109	682	
	3	348	307.09	132.538	7.105	119	1115	
	4	372	393.51	208.041	10.786	104	2498	
	Total	1287	303.47	158.832	4.427	6	2498	
C-reactive protein (mg/L)	1	557	38.1835	54.85315	2.3242	0.4	384.7	< 0.001
	2	564	48.38	67.75575	2.85303	0.4	509.3	
	3	578	69.8327	74.31234	3.09099	0.4	418.6	
	4	577	118.5815	103.7148	4.3177	0.6	569.5	
	Total	2276	69.1298	83.40931	1.74835	0.4	569.5	
Procalcitonin (ng/mL)	1	437	0.31069	2.794236	0.133666	0.02	57.94	0.018
	2	456	0.44145	2.449261	0.114697	0.02	32.54	
	3	487	2.28523	37.79447	1.712631	0.02	831.4	
	4	507	4.27661	21.36995	0.949073	0.03	256.24	
	Total	1887	1.91744	22.28269	0.512958	0.02	831.4	
*Liver enzymes*								
ALT (U/L)	1	422	37.334	34.9598	1.7018	5	273	< 0.001
	2	454	36.455	30.2239	1.4185	4.7	222	
	3	471	44.571	66.076	3.0446	3.5	1091	
	4	508	56.4	127.7525	5.6681	3.2	2666	
	Total	1855	44.178	78.3383	1.8189	3.2	2666	
AST (U/L)	1	75	40.69	48.437	5.593	0	341	< 0.001
	2	86	37.28	40.325	4.348	0	303	
	3	92	46.93	64.371	6.711	12	592	
	4	97	165.62	633.57	64.329	1	5808	
	Total	350	76.12	339.827	18.165	0	5808	
Albumin (g/dL)	1	421	4.0435	0.53899	0.02627	1.8	5.4	< 0.001
	2	454	4.0132	0.51224	0.02404	0.8	5	
	3	469	3.9004	0.52955	0.02445	0.6	5	
	4	507	3.6516	0.53603	0.02381	1.7	4.8	
	Total	1851	3.8925	0.55173	0.01282	0.6	5.4	
Bilirubin, total (mg/dL)	1	421	0.57	0.434	0.021	0	4	0.403
	2	457	0.62	0.631	0.03	0	8	
	3	471	0.71	1.548	0.071	0	31	
	4	507	0.77	0.795	0.035	0	8	
	Total	1856	0.67	0.963	0.022	0	31	
Creatinine (mg/dL)	1	569	1.026	3.8608	0.1619	0.2	92.2	< 0.001
	2	558	0.907	1.0408	0.0441	0.1	24.1	
	3	571	1.284	6.3533	0.2659	0.1	125.9	
	4	578	1.095	1.0308	0.0429	0.2	10.8	
	Total	2276	1.079	3.7931	0.0795	0.1	125.9	

*APTT* activated partial thromboplastin time; *LDH* lactate dehydrogenase; *ALT* alanine aminotransferase; *AST* aspartate aminotransferase

## Discussion

In contrast to the delayed neutrophil response specially in viral infections, circulating monocytes are first responders in a proportional magnitude that match to the intensity of microbial exposure [3]. Blood monocytes are transient stage between site of production and site of action during infection, therefore, assessing monocyte activation by MDW can be a direct measure of the level and stage of infection [7]. MDW is a morphometric biomarker in the course of sepsis development and can be an early indicator of sepsis. Recent studies showed that adding MDW to WBC can enhance medical decision making during early sepsis management especially in neonates patients and whenever monitoring sepsis biomarkers is not accessible due to various reasons such as high coast or testing cannot be done for every suspected cases as in pandemics [5, 8]. Our data showed a significant positive correlation between MDW and various laboratory parameters linked with poor prognosis of COVID-19 including total WBC, neutrophils, liver enzymes and inflammatory markers such as CRP. Furthermore, our data revealed that MDW ≥ 24.685 has a strong correlation with poor prognosis of COVID-19.

A negative correlation between MDW and lymphocytes was noted in the current study which is consistent with several studies’ observations that severe illness is associated with lower lymphocyte counts and may predict poor outcomes and higher rate of mortality in patients with COVID‐19 [9–11]. Studies on SARS suggested that SARS-CoV-2 exhaust and eliminate natural killer cells and T cells leading to lymphopenia, making lymphopenia a useful predictor for prognosis in the patients as Intensive Care Unit (ICU) admitted patients show a dramatic decrease in T cells, especially CD8-T cell counts [12, 13]. Lymphocyte/monocyte count was found to be the main markers discriminating high- and low-risk groups in COVID-19 patients [14]. We found that peripheral blood from deceased patients with COVID-19 frequently showed neutrophilic leukocytosis and lymphopenia that makes serial white blood cell count and lymphocyte count a useful predictors of progression towards a more severe form of COVID-19 as documented by other studies [15, 16]. Additionally, elevated neutrophil counts were significantly correlated to the mortality of COVID‐19 patients, so combined admission lymphopenia and neutrophilia are associated with poor outcomes in patients with COVID-19 [17, 18].

In all cases, the demonstrated correlation between MDW and poor prognostic WBC, neutrophils and lymphocytes is not surprising as previous studies suggested that circulating monocytes and tissue macrophages participate in all stages of SARS COVID-19 [7]. SARS-CoV-2 can infect monocytes through angiotensin-converting enzyme 2(ACE2)-dependent and independent pathways and shifts in monocyte subpopulations in mediating severity of the disease has been proposed [19, 20]. Certain subsets were disturbed and cells co-expressing markers of M1 and M2 monocytes were found in intermediate and non-classical subsets [21]. Those overactivated monocytes play a role in the cytokine storm that leads to the acute pulmonary injury and acute respiratory distress syndrome (ARDS) in COVID‐19 patients [22]. Initially in COVID-19 patients there may be monocytopaenia that is corrected on the 5th day onwards with abnormal activated monocytes characterized by marked anisocytosis, cytoplasmic vacuolisation and paucity of granules [23]. Monocytes in COVID-19 patients have increased lipid droplets accumulation leading to changes in MDW and making this a clinically attractive biomarker for macrophage abnormalities, and structural functional correlation [24].

In our study, MDW was significantly positively correlated with COVID-19 inflammatory markers including CRP, LDH, Ferritin, and Procalcitonin. The level of plasma CRP is known to positively correlate with the severity of COVID-19 pneumonia and can serve as an earlier indicator for severe illness and provides easy guidance to primary care enabling effective intervention measures ahead of time to reduce the rates of severe illness and mortality [25–27]. It is well known that systemic inflammation associated with elevated plasma CRP conferred a phenotype on Peripheral Blood Mononuclear Cells (PBMC), specifically through monocyte tissue factor (TF) expression by monocytes/macrophages leads to thrombin generation linked to sepsis [28, 29]. Moreover, it was reported that monocytes can transport CRP in blood flow through monocyte-derived exosomes to maintain chronic inflammation [30].

The findings of the current study presented significant negative correlation between MDW and total platelet (r = − 0.140, p < 0.001). These findings are concurrent with the fact that COVID-19 is associated with mild thrombocytopenia that is linked with more severe disease and mortality as SARS-CoV-2 can alter platelet number, form, and function [31, 32]. Also, MDW was significantly correlated with the prothrombin time (PT) (r = 0.174, p < 0.001), activated partial thromboplastin time (APTT) (r = 0.204, p < 0.001), and D-Dimer (r = − 0.218, p < 0.001). Studies have reported disturbed coagulation in COVID-19 patients, including decreased antithrombin, prolonged prothrombin time, and increased fibrin degradation products such as D-dimer [33, 34]. This implies increased risk of bleeding, as well as thromboembolic disease that could dispose to the most serious cases including the development of disseminated intravascular coagulation (DIC) [35]. Additionally, D-dimer level at presentation with COVID-19 was shown to predict ICU admission [36].

This study has limitation for being a single-institution study and focused on adults COVID-19 patients. Nevertheless, the interesting about the study is the investigation, for the first time, the correlation between routine laboratory parameters in different quartiles of MDW values and the use of large sample size to support the findings precision. The MDW correlation with different inflammation markers involved in the cytokine storm induced by SARS-CoV-2, such as Interlukin-6 (IL6) and granulocyte colony-stimulating factor (GCSF), is a focal point for future research to increase our understanding of the MDW as a novel sepsis indicator in COVID-19 patients. Further study to investigate the MDW relationship with the clinical evolution of the patients is suggested to make the prognostic value of MDW in disease progress.

## Conclusions

To conclude, MDW can be predictor of poor outcome in patients presenting to the emergency setting with COVID-19. Interventions and specific therapeutics to target macrophage activation may be useful in mitigating adverse outcomes in these populations and manage the inflammatory response in COVID-19, preventing progressing to sepsis and multiorgan failure.

## Supplementary Information

**Additional file 1: Appendix 1.** WHO/International Severe Acute Respiratory and Emerging Infection Consortium case record form for severe acute respiratory infections, which is used to develop data collection form for the current study.

## Data Availability

The datasets generated and/or analyzed during the current study are not publicly available as they form a part of the patients’ medical record at DHA but are available from the corresponding author on reasonable request.

## Abbreviations

MBRU: Mohammed Bin Rashid University of Medicine and Health Sciences
DHA: Dubai Health Authority
DSREC: Dubai Scientific Research Ethics Committee
