# Supplementary material for: Monocyte distribution width as a novel sepsis indicator in COVID-19 patients
Source: BMC Infect Dis. 2022 Jan 4;22:27. doi: 10.1186/s12879-021-07016-4 (PMC8724663; doi:10.1186/s12879-021-07016-4)
Supplement: Supplementary file 1 — Additional file 1: Appendix 1. WHO/International Severe Acute Respiratory and Emerging Infection Consortium case record form for severe acute respiratory infections, which is used to develop data collection form for the current study. [file 12879_2021_7016_MOESM1_ESM.pdf]

World Health Organization ISARIC

COVID-19 CORE CASE REPORT FORM

ACUTE RESPIRATORY INFECTION CLINICAL CHARACTERISATION DATA TOOL

DESIGN OF THIS CASE REPORT FORM (CRF)

This CRF is set up in modules to be used for recording data on the ISARIC_nCov Core Database or for independent studies.

Module 1 and Module 2 complete on the first day of presentation/admission or on first day of COVID-19 assessment.
Module 2 also complete on first day of admission to ICU or high dependency unit. In addition, complete daily for as many days as resources allow up to a maximum of 14 days. Continue to follow-up patients who transfer between wards.
Module 3 (Outcome) complete at discharge or death

GENERAL GUIDANCE

- The CRF is designed to collect data obtained through examination, interview and review of hospital notes. Data may be collected prospectively or retrospectively if the patient is enrolled after the admission date.
- Participant Identification Numbers consist of a 5 digit site code and a 4 digit participant number.
  You can obtain a site code and registering on the data management system by contacting ncov@isaric.org.
  Participant numbers should be assigned sequentially for each site beginning with 0001. In the case of a single site recruiting participants on different wards, or where it is otherwise difficult to assign sequential numbers, it is acceptable to assign numbers in blocks or incorporating alpha characters. E.g. Ward X will assign numbers from 0001 or A001 onwards and Ward Y will assign numbers from 5001 or B001 onwards. Enter the Participant Identification Number at the top of every page.
- Printed paper CRFs may be used for later transfer of the data onto the electronic database.
- For participants who return for re-admission to the same site, **start a new form with the same Participant Identification Number**. Please check “YES-admitted previously” in the ONSET & ADMISSION section. Enter as 2 separate entries in the electronic database.
- For participants who transfer between two sites that are both collecting data on this form, it is preferred to have the data entered by a single site as a single admission, under the same Participant Identification Number. When this is not possible, the first site should record “Transfer to other facility” as an OUTCOME, and the second site should start a new form with a new patient number and indicate “YES-transferred” in ONSET & ADMISSION.
- Complete every line of every section, except where the instructions say to skip a section based on a response.
- Selections with circles (**O**) are single selection answers (choose one answer only). Selections with square boxes (☐) are multiple selection answers (choose as many answers as are applicable).
- Mark ‘Not done’ for any results of laboratory values that are not available, not applicable or unknown.
- Avoid recording data outside of the dedicated areas. Sections are available for recording additional information.
- If using paper CRFs, we recommend writing clearly in ink, using BLOCK-CAPITAL LETTERS.
- Place an (X) when you choose the corresponding answer. To make corrections, strike through (-------) the data you wish to delete and write the correct data above it. Please initial and date all corrections.
- Please keep all of the sheets for a single participant together e.g. with a staple or participant-unique folder.
- Please transfer all paper CRF data to the electronic database. All paper CRFs needs to be stored locally, do not send any forms to us. Data are accepted only via secure electronic database.
- Please enter data on the electronic data capture system at https://ncov.medsci.ox.ac.uk/. If your site would like to collect data independently, we are happy to support the establishment of locally hosted databases.
- Please contact us at ncov@isaric.org if you need help with databases, if you have comments and to let us know that you are using the forms.

Adapted from SPRINT SARI CRF by ISARIC. Licensed under a Creative Commons Attribution-ShareAlike 4.0 International License by ISARIC on behalf of Oxford University.

World Health Organization ISARIC

PARTICIPANT IDENTIFICATION #: [___][___][___][___][___]--- [___][___][___][___]

MODULE 1: PRESENTATION/ADMISSION CASE REPORT FORM

CLINICAL INCLUSION CRITERIA

Suspected or confirmed novel coronavirus (COVID-19) infection: ○YES ○NO

DEMOGRAPHICS

Clinical centre name:_______________________________________ **Country:**_______________________________________

Enrolment date /first COVID-19 assessment date: [_D_][_D_]/[_M_][_M_]/[_2_][_0_][_Y_][_Y_]

Ethnic group *(check all that apply)*: ☐Arab ☐Black ☐East Asian ☐South Asian ☐ West Asian ☐Latin American ☐White

☐Aboriginal/First Nations ☐Other: ________________________ ○Unknown

Employed as a Healthcare Worker? ○YES ○NO ○Unknown **Employed in a microbiology laboratory?** ○YES ○NO ○Unknown

Sex at Birth: ○Male ○Female ○Not specified/Unknown **Age** [___][___][___]years OR [___][___]months

Pregnant? ○YES ○NO ○Unknown **If YES: Gestational weeks assessment:** [___][___] weeks

POST PARTUM? ○YES ○NO ○Unknown *(if NO or Unknown skip this section)*

Pregnancy Outcome: ○Live birth ○Still birth **Delivery date:** [_D_][_D_]/[_M_][_M_]/[_2_][_0_][_Y_][_Y_]

Baby tested for COVID-19/SARS-CoV-2 infection? ○YES ○NO ○Unknown

If YES, result of test: ○Positive ○Negative ○Unknown (If Positive, complete a separate CRF for baby)

INFANT – Less than 1 year old? ○YES ○NO *(If NO skip this section)*

Birth weight: [___][___].[___]○kg or ○lbs ○Unknown

Gestational outcome: ○ Term birth (≥37wk GA) ○Preterm birth (<37wk GA) ○Unknown

Breastfed? ○YES-currently breastfeeding ○YES-breastfeeding discontinued ○NO ○Unknown

Vaccinations appropriate for age/country? ○YES ○NO ○Unknown

ONSET & ADMISSION

Onset date of first/earliest symptom: [_D_][_D_]/[_M_][_M_]/[_2_][_0_][_Y_][_Y_]

Most recent presentation/admission date at this facility: [_D_][_D_]/[_M_][_M_]/[_2_][_0_][_Y_][_Y_]

Was the patient admitted previously or transferred from any other facility during this illness episode?

○YES-admitted previously to this facility ○YES–transferred from other facility ○NO ○Unknown

SIGNS AND SYMPTOMS AT HOSPITAL ADMISSION *(first available data at presentation/admission – within 24 hours)*

Temperature: [____][____][____].[____]○°C *or* ○°F

HR: [____][____][____]beats per minute **RR:** [____][____]breaths per minute

Systolic BP: [___][___][___]mmHg **Diastolic BP:** [____][____][____]mmHg

Oxygen saturation: [___][___][___]% **On:** ○Room air ○Oxygen therapy ○Unknown

Sternal capillary refill time >2sec. ○YES ○NO ○Unknown **Height:** [___][___][___]cm **Weight:** [___][___][___]kg

Adapted from SPRINT SARI CRF by ISARIC. Licensed under a Creative Commons Attribution-ShareAlike 4.0 International License by ISARIC on behalf of Oxford University.

World Health Organization ISARIC

PARTICIPANT IDENTIFICATION #: [___][___][___][___][___]--- [___][___][___][___]

MODULE 1: PRESENTATION/ADMISSION CASE REPORT FORM

**SIGNS AND SYMPTOMS ON ADMISSION** *(Unk = Unknown)*			
History of fever	❍YES ❍NO ❍Unk	Fatigue / Malaise	❍YES ❍NO ❍Unk
Cough ❍YES-non-productive ❍YES-productive ❍YES-with haemoptysis ❍NO ❍Unk		Anorexia	❍YES ❍NO ❍Unk
		Altered consciousness/confusion	❍YES ❍NO ❍Unk
Sore throat	❍YES ❍NO ❍Unk	Muscle aches (myalgia)	❍YES ❍NO ❍Unk
Runny nose (rhinorrhoea)	❍YES ❍NO ❍Unk	Joint pain (arthralgia)	❍YES ❍NO ❍Unk
Wheezing	❍YES ❍NO ❍Unk	Inability to walk	❍YES ❍NO ❍Unk
Shortness of breath	❍YES ❍NO ❍Unk	Abdominal pain	❍YES ❍NO ❍Unk
Lower chest wall indrawing	❍YES ❍NO ❍Unk	Diarrhoea	❍YES ❍NO ❍Unk
Chest pain	❍YES ❍NO ❍Unk	Vomiting / Nausea	❍YES ❍NO ❍Unk
Conjunctivitis	❍YES ❍NO ❍Unk	Skin rash	❍YES ❍NO ❍Unk
Lymphadenopathy	❍YES ❍NO ❍Unk	Bleeding (Haemorrhage)	❍YES ❍NO ❍Unk
Headache	❍YES ❍NO ❍Unk	If YES, specify site(s):	
Loss of smell (Anosmia)	❍YES ❍NO ❍Unk	Other symptom(s)	❍YES ❍NO ❍Unk
Loss of taste (Ageusia)	❍YES ❍NO ❍Unk	If YES, specify:	
Seizures	❍YES ❍NO ❍Unk		

**PRE-ADMISSION MEDICATION** *(taken within 14 days of admission/presentation at healthcare facility)*	
Angiotensin converting enzyme inhibitors (ACE inhibitors)	❍YES ❍NO ❍Unk
Angiotensin II receptor blockers (ARBs)	❍YES ❍NO ❍Unk
Non-steroidal anti-inflammatory (NSAIDs)	❍YES ❍NO ❍Unk
Oral steroids	❍YES ❍NO ❍Unk If YES, agent(s):
Other immunosuppressant agents (not oral steroids)	❍YES ❍NO ❍Unk If YES, agent(s):
Antivirals	❍YES ❍NO ❍Unk If YES, agent(s):
Antibiotics	❍YES ❍NO ❍Unk If YES, agent(s):
Other targeted COVID-19 Medications	❍YES ❍NO ❍Unk If YES, agent(s):

**CO-MORBIDITIES AND RISK FACTORS** *(existing prior to admission and ongoing)*			
Chronic cardiac disease *(not hypertension)*	❍YES ❍NO ❍Unk	Chronic hematologic disease	❍YES ❍NO ❍Unk
Hypertension	❍YES ❍NO ❍Unk	AIDS / HIV ❍YES-on ART ❍YES-not on ART ❍NO ❍Unk	
Chronic pulmonary disease *(not asthma)*	❍YES ❍NO ❍Unk	Diabetes Mellitus ❍YES-Type 1 ❍YES -Type 2 ❍NO ❍Unk	
Asthma *(physician diagnosed)*	❍YES ❍NO ❍Unk	Rheumatologic disorder	❍YES ❍NO ❍Unk
Chronic kidney disease	❍YES ❍NO ❍Unk	Dementia	❍YES ❍NO ❍Unk
Obesity *(as defined by clinical staff)*	❍YES ❍NO ❍Unk	Tuberculosis	❍YES ❍NO ❍Unk
Moderate or severe liver disease	❍YES ❍NO ❍Unk	Malnutrition	❍YES ❍NO ❍Unk
Mild liver disease	❍YES ❍NO ❍Unk	Smoking ❍YES ❍Never smoked ❍Former smoker ❍Unk	
Asplenia	❍YES ❍NO ❍Unk	Other relevant risk factor(s)	❍YES ❍NO ❍Unk
Chronic neurological disorder	❍YES ❍NO ❍Unk	If YES, specify:	
Malignant neoplasm	❍YES ❍NO ❍Unk		

Adapted from SPRINT SARI CRF by ISARIC. Licensed under a Creative Commons Attribution-ShareAlike 4.0 International License by ISARIC on behalf of Oxford University.

MODULE 2: DAILY CASE REPORT FORM

Complete on the day of admission or first COVID-19 investigation, and on the first day of ICU admission (if different from day of admission). In addition, depending on available resources, complete every day for a maximum of 14 days, or for days when biochemical results are available.

SIGNS AND SYMPTOMS *(Record the worst value between 00:00 to 24:00 on day of assessment)(worst=furthest from normal range)*

DATE OF ASSESSMENT *(DD/MM/YYYY):* [_D_][_D_]/[_M_][_M_]/[__2_][__0_][_Y_][_Y_]

Temperature: [___][___].[___] ○°C *or* ○°F **HR:** [___][___][___]beats per minute **RR:** [___][___][___]breaths per minute

Systolic BP: [___][___][___]mmHg **Diastolic BP:** [___][___][___]mmHg **Oxygen saturation SaO_2** [___][___][___]%

Any supplemental oxygen: FiO_2 *(0.21-1.0)* [___].[___][___] or [___][___] % or [___][___]L/min

Sternal capillary refill time >2seconds ○YES ○NO ○Unknown

AVPU: Alert [___] Verbal[___] Pain [___] Unresponsive [___] **Glasgow Coma Score (GCS / 15)** [___][___]

Is the patient currently receiving, or has received *(between 00:00 to 24:00 on day of assessment)*

High-flow nasal cannula oxygen therapy? ○YES ○NO ○Unknown

Non-invasive ventilation *(Any)*? ○YES ○NO ○Unknown **If YES:** ○BIPAP ○CPAP ○Other ○Unknown

Invasive ventilation? ○YES ○NO ○Unknown

Prone positioning? ○YES ○NO ○Unknown

Inhaled Nitric Oxide? ○YES ○NO ○Unknown

Tracheostomy inserted? ○YES ○NO ○Unknown

Extra corporeal life support (ECLS/ ECMO)? ○YES ○NO ○Unknown **If YES:** ○VV ○AV ○Central ○Unknown

Renal replacement therapy (RRT) or dialysis? ○YES ○NO ○Unknown

Any vasopressor/inotropic support? ○YES ○NO ○Unknown *(if NO, select NO for the next 3 questions)*

- **Dopamine <5µg/kg/min OR Dobutamine OR milrinone OR levosimendan:** ○YES ○NO
- **Dopamine 5-15µg/kg/min OR Epinephrine/Norepinephrine < 0.1µg/kg/min OR vasopressin OR phenylephrine**: ○YES ○NO
- **Dopamine >15µg/k/min OR Epinephrine/Norepinephrine > 0.1µg/kg/min**: ○YES ○NO

Neuromuscular blocking agents? ○YES ○NO ○Unknown

Other intervention(s) or procedure(s)? ○YES ○NO ○Unknown If YES, Specify: ______________________________

____

Current admission to ICU/ITU/IMC/HDU? ○YES ○NO ○Unknown ***(Record the worst value on day of assessment)***

PaO_2 *(at time nearest to the FiO_2 recorded at top of page)* [___][___][___]○kPa *or* ○mmHg ○Not done

PaO_2 sample type: ○Arterial ○Capillary ○Unknown

From same blood gas record as PaO_2:

PCO_2 ____________○kPa *or* ○mmHg | **pH** _____________ | **HCO_3-** ___________mEq/L | **Base excess** __________ mmol/L

Richmond Agitation-Sedation Scale (RASS) [___] or **Riker Sedation-Agitation Scale (SAS)** [___] ○Unknown

Mean Arterial Blood Pressure [___][___][___]mmHg ○Unknown

Urine flow rate [___][___][___][___][___]mL/24 hours ○ Check if estimated ○Unknown

Adapted from SPRINT SARI CRF by ISARIC. Licensed under a Creative Commons Attribution-ShareAlike 4.0 International License by ISARIC on behalf of Oxford University.

MODULE 2: DAILY CASE REPORT FORM

Complete on the day of admission or first COVID-19 investigation, and on the first day of ICU admission (if different from day of admission). In addition, depending on available resources, complete every day for a maximum of 14 days, or for days when biochemical results are available.

LABORATORY RESULTS (on admission, on any admission to ICU, then daily) – complete every line

DATE OF ASSESSMENT *(DD/MM/YYYY):* [_D_][_D_]/[_M_][_M_]/[_2_][_0_][_Y_][_Y_]

Record the worst value between 00:00 to 24:00 on day of assessment (if Not Available write 'N/A'):

LABORATORY RESULTS *(*record units if different from those listed)*

Parameter	Value*	Not done	Parameter	Value*	Not done
Haemoglobin (g/L)		○	Urea (BUN) (mmol/L)		○
WBC count (x10^9/L)		○	Lactate (mmol/L)		○
Lymphocyte count (10^9/L)		○	Creatinine (μmol/L)		○
Neutrophil count (10^9/L)		○	Sodium (mmol/L)		○
Haematocrit (%)		○	Potassium (mmol/L)		○
Platelets (x10^9/L)		○	Procalcitonin (ng/mL)		○
APTT (seconds))		○	CRP (mg/L)		○
APTR		○	LDH (U/L)		○
PT (seconds)		○	Creatine kinase (U/L)		○
INR		○	Troponin I (ng/mL)		○
ALT/SGPT (U/L)		○	D-dimer (mg/L)		○
Total bilirubin (μmol/L)		○	Ferritin (ng/mL)		○
AST/SGOT (U/L)		○	IL-6 (pg/mL)		○
Glucose (mmol/L)		○			

Adapted from SPRINT SARI CRF by ISARIC. Licensed under a Creative Commons Attribution-ShareAlike 4.0 International License by ISARIC on behalf of Oxford University.

MODULE 3: OUTCOME CASE REPORT FORM

TREATMENT: At ANY time during hospitalisation, did the patient receive/undergo:

Any Oxygen therapy? OYES ONO OUnknown **If YES, total duration:** _________days OUnknown

Maximum O_2 flow volume: O <2 L/min O2-5 L/min O6-10 L/min O11-15 L/min O>15 L/min

Non-invasive ventilation? *(Any)* OYES ONO OUnknown **If YES, total duration:** _________days OUnknown

Invasive ventilation? *(Any)* OYES ONO OUnknown **If YES, total duration:** _________days OUnknown

Prone Positioning? OYES ONO OUnknown **If YES, total duration:** _________days OUnknown

Inhaled Nitric Oxide? OYES ONO OUnknown

Tracheostomy inserted? OYES ONO OUnknown

Extracorporeal support (ECMO)? OYES ONO OUnknown **If YES, total duration:** _________days OUnknown

Renal replacement therapy (RRT) or dialysis? OYES ONO OUnknown

Inotropes/vasopressors? OYES ONO OUnknown **If YES, total duration:** _________days OUnknown

ICU or High Dependency Unit admission? OYES ONO OUnknown **If YES, total duration:** _________days OUnknown

If YES, date of ICU admission: [_D_][_D_]/[_M_][_M_]/[_2_][_0_][_Y_][_Y_] OUnknown

date of ICU discharge: [_D_][_D_]/[_M_][_M_]/[_2_][_0_][_Y_][_Y_] OUnknown

COMPLICATIONS: At any time during hospitalisation did the patient experience: *(Unk = Unknown)*

Viral pneumonia/pneumonitis	OYES ONO OUnk	Stroke / Cerebrovascular accident	OYES ONO OUnk
Bacterial pneumonia	OYES ONO OUnk	Meningitis / Encephalitis	OYES ONO OUnk
Acute Respiratory Distress Syndrome	OYES ONO OUnk	Bacteremia	OYES ONO OUnk
If YES, specify: O Mild O Moderate O Severe OUnk		Coagulation disorder / DIC	OYES ONO OUnk
Pneumothorax	OYES ONO OUnk	Pulmonary embolism	OYES ONO OUnk
Pleural effusion	OYES ONO OUnk	Anemia	OYES ONO OUnk
Cryptogenic organizing pneumonia (COP)	OYES ONO OUnk	Rhabdomyolysis / Myositis	OYES ONO OUnk
Bronchiolitis	OYES ONO OUnk	Acute renal injury/ Acute renal failure	OYES ONO OUnk
Cardiac arrest	OYES ONO OUnk	Gastrointestinal haemorrhage	OYES ONO OUnk
Myocardial infarction	OYES ONO OUnk	Pancreatitis	OYES ONO OUnk
Cardiac ischaemia	OYES ONO OUnk	Liver dysfunction	OYES ONO OUnk
Cardiac arrhythmia	OYES ONO OUnk	Hyperglycemia	OYES ONO OUnk
Myocarditis / Pericarditis	OYES ONO OUnk	Hypoglycemia	OYES ONO OUnk
Endocarditis	OYES ONO OUnk	Other	
Cardiomyopathy	OYES ONO OUnk	If YES specify:	
Congestive heart failure	OYES ONO OUnk		
Seizure	OYES ONO OUnk		

Adapted from SPRINT SARI CRF by ISARIC. Licensed under a Creative Commons Attribution-ShareAlike 4.0 International License by ISARIC on behalf of Oxford University.

World Health Organization ISARIC

PARTICIPANT IDENTIFICATION #: [___][___][___][___][___]--- [___][___][___][___]

MODULE 3: OUTCOME CASE REPORT FORM

DIAGNOSTICS

Was patient clinically diagnosed with COVID-19? ○YES ○NO ○Unknown

Was pathogen testing done during this illness episode? ○YES *(complete section)* ○NO ○Unknown

Coronavirus: ○Positive ○Negative ○Not done **If Positive:** ○COVID-2019/ SARS-CoV2 ○MERS CoV ○Other CoV: ____________________ ○Unknown

Influenza : ○Positive ○Negative ○Not done **If Positive:** ○A/H3N2 ○A/H1N1pdm09 ○A/H7N9 ○A/H5N1 ○A-not typed ○B ○Other:__________________________ ○Unknown

RSV: ○Positive ○Negative ○Not done

Adenovirus: ○Positive ○Negative ○Not done

Bacteria: ○Positive ○Negative ○Not done **If Positive, specify**: _________________ _________________ ○Unknown

Other pathogen/s detected: ○YES ○NO ○Unknown **If YES, specify all:**_________________________________○Unknown

****

Clinical pneumonia diagnosed? ○YES ○NO ○Unknown

Chest X-Ray performed? ○YES ○NO ○Unknown **If Yes: Were infiltrates present?** ○YES ○NO ○Unknown

CT performed? ○YES ○NO ○Unknown **If Yes: Were infiltrates present?** ○YES ○NO ○Unknown

Collection Date *(DD/MM/YYYY)*	Biospecimen Type	Laboratory test Method	Result	Pathogen Tested/Detected
_D_ _D_ /_M_ _M_ /20_Y_ _Y_	○Nasal/NP swab ○Throat swab<br>○Combined nasal/NP+throat swab<br>○Sputum ○BAL ○ETA ○Urine<br>○Feces/rectal swab ○Blood<br>○Other, *Specify:* ____________________	○PCR<br>○Culture<br>○Other, *Specify:*<br>______________	○Positive<br>○Negative<br>○Unknown	________________
_D_ _D_ /_M_ _M_ /20_Y_ _Y_	○Nasal/NP swab ○Throat swab<br>○Combined nasal/NP+throat swab<br>○Sputum ○BAL ○ETA ○Urine<br>○Feces/rectal swab ○Blood<br>○Other, *Specify:* ____________________	○PCR<br>○Culture<br>○Other, *Specify:*<br>______________	○Positive<br>○Negative<br>○Unknown	________________
_D_ _D_ /_M_ _M_ /20_Y_ _Y_	○Nasal/NP swab ○Throat swab<br>○Combined nasal/NP+throat swab<br>○Sputum ○BAL ○ETA ○Urine<br>○Feces/rectal swab ○Blood<br>○Other, *Specify:* ____________________	○PCR<br>○Culture<br>○Other, *Specify:*<br>______________	○Positive<br>○Negative<br>○Unknown	________________
_D_ _D_ /_M_ _M_ /20_Y_ _Y_	○Nasal/NP swab ○Throat swab<br>○Combined nasal/NP+throat swab<br>○Sputum ○BAL ○ETA ○Urine<br>○Faeces/rectal swab ○Blood<br>○Other, *Specify:* ____________________	○PCR<br>○Culture<br>○Other, *Specify:*<br>______________	○Positive<br>○Negative<br>○Unknown	________________
_D_ _D_ /_M_ _M_ /20_Y_ _Y_	○Nasal/NP swab ○Throat swab<br>○Combined nasal/NP+throat swab<br>○Sputum ○BAL ○ETA ☐Urine<br>○Feces/rectal swab ○Blood<br>○Other, *Specify:* ____________________	○PCR<br>○Culture<br>○Other, *Specify:*<br>______________	○Positive<br>○Negative<br>○Unknown	________________

Adapted from SPRINT SARI CRF by ISARIC. Licensed under a Creative Commons Attribution-ShareAlike 4.0 International License by ISARIC on behalf of Oxford University.

MODULE 3: OUTCOME CASE REPORT FORM

MEDICATION: While hospitalised or at discharge, were any of the following administered? *(Unk=Unknown)*

Antiviral or COVID-19 targeted agent? ○YES ○NO ○Unknown **If YES, specify all agents and duration:**

☐**Ribavirin** **Date commenced** [_D_][_D_]/[_M_][_M_]/[_2_][_0_][_Y_][_Y_] **Duration**: _______ days ○Unk

☐ **Lopinavir/Ritonavir** **Date commenced** [_D_][_D_]/[_M_][_M_]/[_2_][_0_][_Y_][_Y_] **Duration**: _______ days ○Unk

☐**Remdesivir** **Date commenced** [_D_][_D_]/[_M_][_M_]/[_2_][_0_][_Y_][_Y_] **Duration**: _______ days ○Unk

☐**Interferon alpha** **Date commenced** [_D_][_D_]/[_M_][_M_]/[_2_][_0_][_Y_][_Y_] **Duration**: _______ days ○Unk

☐**Interferon beta** **Date commenced** [_D_][_D_]/[_M_][_M_]/[_2_][_0_][_Y_][_Y_] **Duration**: _______ days ○Unk

☐ **Chloroquine/hydroxychloroquine** **Date commenced** [_D_][_D_]/[_M_][_M_]/[_2_][_0_][_Y_][_Y_] **Duration**: _______days ○Unk

☐**Other** _____________________ **Date commenced** [_D_][_D_]/[_M_][_M_]/[_2_][_0_][_Y_][_Y_] **Duration**: _______days ○Unk

****

Antibiotic? ○YES ○NO ○Unk **If yes, specify all:**

Agent:______________________ **Date commenced** [_D_][_D_]/[_M_][_M_]/[_2_][_0_][_Y_][_Y_] **Duration:** ____ days ○Unk

Agent:______________________ **Date commenced** [_D_][_D_]/[_M_][_M_]/[_2_][_0_][_Y_][_Y_] **Duration:** ____ days ○Unk

Agent:______________________ **Date commenced** [_D_][_D_]/[_M_][_M_]/[_2_][_0_][_Y_][_Y_] **Duration:** ____ days ○Unk

****

Corticosteroid? ○YES ○NO ○Unk **If YES, Route:** ☐Oral ☐Intravenous (IV) ☐Inhaled ○Unk

If YES Oral or IV, please provide agent: ___________________________________ **and max. daily dose & unit:**_____________

Date commenced [_D_][_D_]/[_M_][_M_]/[_2_][_0_][_Y_][_Y_] ○Unk **Duration**: _______ days ○Unk

****

Heparin? ○YES ○NO ○Unk **If YES, Route:** ☐Subcutaneous ☐Intravenous (IV) ○Unk

If YES: ☐Unfractionated ☐Low molecular weight ☐Fondaparinux ○Unk **Maximum daily dose & unit:**________________

Date commenced [_D_][_D_]/[_M_][_M_]/[_2_][_0_][_Y_][_Y_] ○Unk **Duration**: _______ days ○Unk

****

Antifungal agent? ○YES ○NO ○Unk

****

Other treatments administered for COVID-19 including experimental or compassionate use? ○YES ○NO ○Unk

If yes, specify agent, maximum daily does and duration:

Agent: __________________________ **Maximum daily dose & unit**: ________________________ ○Unk

Date of commencement [_D_][_D_]/[_M_][_M_]/[_2_][_0_][_Y_][_Y_] ○Unk **Duration**: _______ days ○Unk

Agent:__________________________ **Maximum daily dose & unit**: _________________________ ○Unk

Date of commencement [_D_][_D_]/[_M_][_M_]/[_2_][_0_][_Y_][_Y_] ○Unk **Duration**: _______ days ○Unk

OUTCOME

Outcome: ○Discharged alive ○Hospitalised ○Transfer to other facility ○Death ○Palliative discharge ○Unknown

Outcome date: [_D_][_D_]/[_M_][_M_]/[_2_][_0_][_Y_][_Y_] ○Unknown

If Discharged alive:

Ability to self-care at discharge versus before illness: ○Same as before illness ○Worse ○Better ○Unknown

Post-discharge treatment: Oxygen therapy? ○YES ○NO ○Unknown

Adapted from SPRINT SARI CRF by ISARIC. Licensed under a Creative Commons Attribution-ShareAlike 4.0 International License by ISARIC on behalf of Oxford University.
